# Petroleum-Degrading Fungal Isolates for the Treatment of Soil Microcosms

**DOI:** 10.3390/microorganisms11051351

**Published:** 2023-05-22

**Authors:** Dalel Daâssi, Fatimah Qabil Almaghrabi

**Affiliations:** 1 Laboratory of Environmental Bioprocesses, Centre of Biotechnology of Sfax, P.O. Box 1177, Sfax 3018, Tunisia; 2 Department of Biology, College of Sciences and Arts, Khulais, University of Jeddah, P.O. Box 34, Jeddah 21959, Saudi Arabia; falmaghrabi1@gmail.com

**Keywords:** single culture, fungal consortium, bioaugmentation, GC-MS, PAH-contaminated soil

## Abstract

The main purpose of this study was to degrade total petroleum hydrocarbons (TPHs) from contaminated soil in batch microcosm reactors. Native soil fungi isolated from the same petroleum-polluted soil and ligninolytic fungal strains were screened and applied in the treatment of soil-contaminated microcosms in aerobic conditions. The bioaugmentation processes were carried out using selected hydrocarbonoclastic fungal strains in mono or co-cultures. Results demonstrated the petroleum-degrading potential of six fungal isolates, namely KBR1 and KBR8 (indigenous) and KBR1-1, KB4, KB2 and LB3 (exogenous). Based on the molecular and phylogenetic analysis, KBR1 and KB8 were identified as *Aspergillus niger* [MW699896] and *tubingensis* [MW699895], while KBR1-1, KB4, KB2 and LB3 were affiliated with the genera *Syncephalastrum* sp. [MZ817958], *Paecilomyces formosus* [MW699897], *Fusarium chlamydosporum* [MZ817957] and *Coniochaeta* sp. [MW699893], respectively. The highest rate of TPH degradation was recorded in soil microcosm treatments (SMT) after 60 days by inoculation with *Paecilomyces formosus* 97 ± 2.54%, followed by bioaugmentation with the native strain *Aspergillus niger* (92 ± 1.83%) and then by the fungal consortium (84 ± 2.21%). The statistical analysis of the results showed significant differences.

## 1. Introduction

Over the last two decades, accelerated industrialisation and the massive use of aromatic compounds in explosives, dyestuffs, pesticides and pharmaceuticals have resulted in serious environmental contamination of the soil, water and air. Oil spillage is a serious threat to all components of the ecosystem. During extraction, transportation, storage and distribution operations, crude oil and its refined products are frequently exposed to accidental spillage, causing soil pollution [1].

Soils contaminated with persistent organic pollutants (POPs), associated with petroleum, have high potential health risks because of their ability to enter the food chain and their affinity for accumulation in living organisms [2]. Soil matrix properties and functions are closely related to different activities occurring on land and xenobiotic structures such as Petroleum Hydrocarbons (PHs). Owing to the chemical stability of PHs, their hydrophobicity and their recalcitrance to microbial degradation, spilled oil may damage the biological and physicochemical properties of the petroleum-polluted soil. Petroleum hydrocarbons cause the alteration of soil biological properties, which affects microbial diversity and enzymatic activities as well as physicochemical characteristics [3,4]. Certain essential soil functions may be lost due to the high toxicity of such persistent aromatic hydrocarbon structures. Indeed, spilled oil may develop anaerobic conditions and asphyxia in soil pores with consequent impacts on microbial activities [5]. In this regard, Klamerus-Iwan et al. [6] demonstrated a significant decline in the microbial biomass and enzymatic activities (urease and dehydrogenase) of soil polluted by chainsaw oil. Furthermore, petroleum-polluted areas are characterised by a lower self-purification capacity, which results in the reduction of indigenous microbes involved in soil-purification processes [7].

The remediation of petroleum-polluted soils is important to remove pollutants from the environment, and it can be carried out with several different methods involving the removal/isolation or alteration of the contaminant. In this regard, various physicochemical treatment techniques for soil reclamation have been extensively tested to remove or transform PHs pollutants. Some of these remediation techniques are plasma oxidation, photocatalytic degradation, vapor extraction, flotation, ultrasonication, electrokinetic remediation, thermal desorption and biochar adsorption [8,9,10]. However, those strategies are costly and most of them are not completely effective. As an alternative, biological treatment, or bioremediation, has become a promising approach for restoring petroleum-contaminated regions. Generally, the biological method employs the natural potential of microbes, including bacteria and yeasts (bacterial remediation), algae (phycoremediation) and fungi (mycoremediation), for the biodegradation of petroleum hydrocarbon pollutants.

Bioremediation effectiveness is often related to the microbial population and how it can be enriched and maintained in polluted areas [11].

The simplest bioremediation strategy is natural attenuation. This method requires only the control of the natural degradation processes occurring in the native microbial population. However, this approach is not always successful and requires extensive, long-term monitoring. It can be used for the restoration of areas with low levels of contamination. Approximately 25% of all petroleum-contaminated land has been remediated using natural attenuation [12]. Conventionally, bioremediation processes can be accelerated by enhancing intrinsic microbial populations with potentially pollutant-degrading microbes (either indigenous or exogenous microorganisms). This approach is often used for high concentrations of spilled oil in which natural degrading microbes are absent or insufficient [13]. Indeed, hydrocarbon compounds can delay or inhibit microbial proliferation and activities, so for effective in situ biodegradation bioaugmentation is important [14]. The employment of an indigenous microorganism consortium ensures that the organisms have a higher tolerance to the toxicity of aromatic hydrocarbons and are resistant to variations in the environment [15,16]. Exogenous microbes are useful in more complex hydrocarbon structures wherein the rate of intrinsic biodegradation is slower than hydrocarbon degradation. Therefore, bioaugmentation approaches are necessary to enhance the performance of the indigenous microbial population severalfold through the introduction of microbes, with specific metabolic activities for the effective in situ remediation of polluted areas [17].

Typically, fungi are suited for the bioremediation of crude oil in polluted sites owing to their diverse metabolic activities. They can secrete a broad range of ligninolytic and non-ligninolytic enzymes to use petroleum hydrocarbons as carbon and energy sources, and assimilate them into the fungal biomass [18]. Moreover, the efficiency of the fungal culture in removing or degrading hydrocarbons from petroleum-contaminated soil is related to various factors, such as pollutant bioavailability, the survival of microorganisms and their metabolic diversity, which are essential for bioaugmentation [19]. Previously, it has been demonstrated that soil microcosms (SMs) can serve as test systems that may be adapted to various environmental conditions. Indeed, the outcomes of microcosm studies are often used to develop remedial pilot process specifications [20].

According to early considerations, oil-polluted areas are favourable to the proliferation of indigenous microbial populations well adapted to degrade and use petroleum as a source of carbon, and may be used as a potential resource for remediation. Additionally, in the literature, wood-decomposing fungi are well recognised as efficient PHs-degraders due to the action offered by their ligninolytic and non-ligninolytic enzymatic system [18].

Regarding biotechnological applications, the isolation and identification of new fungal PHs-degrader strains are of great interest. The current study highlights the application of new fungal isolates (indigenous from the investigated soil and exogenous from wood) in petroleum-contaminated soil remediation processes. Individual and mixed fungal cultures were selected based on their taxonomy and metabolic diversity to enhance the removal of TPH in soil microcosm systems.

## 2. Materials and Methods

### 2.1. Chemicals

All chemicals used were analytical grade and acquired from Sigma-Aldrich (St. Louis, MO, USA).

### 2.2. Decaying Wood Sampling

Pieces of wood debris were collected in the period of March 2020 from Barzah and Rahat in the Khulais region, Jeddah City, Saudi Arabia. Samples were transported to the laboratory in sterilised and labelled bags with specific data, such as location, number and relevant characteristics.

### 2.3. Soil Sampling and Characterisation

Petroleum-contaminated soil samples were collected from spots around oil well 7, located in Dammam City, Saudi Arabia. All samples were taken at a depth of 5–10 cm from the upper surface of the topsoil. The samples were transferred to the laboratory in sterilised nylon sacks, closed tightly and marked with the relevant information (number, location-specific characteristics and date). Before utilisation in the treatment study, all soil samples were mixed and sieved with a 6 mm grid, then stored under 4 °C. The physicochemical analysis was performed by Arabia Life Sciences Division-Environmental Saudi Arabia (ALS), which is a diversified testing service organisation.

ALS uses analytical procedures developed from established, internationally recognised procedures, such as those published by the USEPA, APHA AS, NEPM, FDA/BAM, AOAC and ISO (Supplementary Materials Table S1).

The pH, moisture content and total petroleum hydrocarbons (TPH), consisting of semi-volatile and volatile organic compounds and the benzene, toluene, ethylbenzene and xylene (BTEX) levels of the petroleum-contaminated soil, were determined before and after the fungal treatments.

For the estimation of the semi-volatile TPH, the USEPA 8015B method of gas chromatography/flame ionisation detection (GC/FID) was conducted. The sample extracts were analysed using capillary GC/FID and quantified against alkane standards over the range of C10–C40.

Volatile TPH/BTEX was determined using the method of EPA 8260 purge-and-trap gas chromatography–mass spectrometry (GC-MS). The extracts were analysed with purge-and-trap capillary GC-MS. The methanol extraction of the soil for purge and trap was performed using the USEPA SW 846–5030A method.

The soil pH was determined by a digital pH meter in a soil–water suspension (1:2.5), as described by Jackson [21].

The moisture content of the soil was determined gravimetrically, based on weight loss after a 12 h drying period at 103–105 °C. This method was compliant with the National Environment Protection Measure (NEPM) [22].

### 2.4. Isolation of Wood-Decay Decomposing Fungi and Indigenous Soil Fungi

Decayed wood samples were collected in sterilised and labelled plastic bags from the Barzah and Rahat regions of Khulais, Jeddah City, Saudi Arabia, in the period March 2020. Malt extract agar (MEA) (30.0 g L^−1^, pH 5.5) supplemented with antibiotics (0.01% of ampicillin and streptomycin) was used as the selective media for the isolation of fungal strains. The isolation of wood-decay decomposing fungi was carried out by the direct plate method suggested by Daâssi et al. [23]. The purity of the fungal strain was proven by microscopic observation.

Both the soil-plate and soil-suspension methods were used for the isolation of the soil fungi [24]. The plates were prepared by transferring 0.05–0.015 g of contaminated soil to a sterilised Petri dish to be examined.

A cooling medium of MEA was added, and the soil particles were dispersed throughout the agar by gently shaking the plates before the agar solidified. A soil-suspension solution was prepared from 10 g of dried contaminated soil dissolved in 100 mL of sterile physiological water (NaCl 9 g L^−1^) and maintained under agitation for 20 min at 120 rpm. After shaking, the serial dilution and plating techniques were performed according to Agrawal et al. [25].

All plates were incubated at 30 °C for five to seven days. The fungal colony was subcultured on fresh MEA supplemented with 0.01% of (Penicillin/streptomycin) solution (gibco by life technology) until a pure strain was obtained. The preliminary identification of the fungal isolates was performed through macroscopic and microscopic observation.

### 2.5. Selection of Hydrocarbon-Degrading Fungal Isolates

The preliminary screening of the oil-degrading fungal isolates (both ligninolytic and native fungal isolates) was performed using the agar well diffusion method and in flasks culture. As the sole source of carbon and energy, 5% crude oil (Saudi Aramco, Jeddah, Saudi Arabia), defined according to Daâssi et al. [26], was spread on the surface of the MEA plates with a glass rod or added to culture flasks. Fungal strain suspensions (in sterile water) were prepared and downloaded in MEA plate wells. The culture plates were incubated at 30 °C, and the appearance of substantial growth was monitored daily for five days. Furthermore, the culture plates with and without the addition of diesel fuel were examined for growth. Before autoclaving, the pH of the solution was adjusted. Then, 5% crude oil was mixed with 100 mL of MM and inoculated with 1% of the conidia suspension of each isolate. Then, 250-mL Erlenmeyer flasks were incubated under shaking (30 °C, 150 rpm) for 14 days at 30 °C.

### 2.6. Molecular Identification and Phylogenetic Tree of Fungal Isolates

The selected hydrocarbon-degrading fungal strains were cultivated in a 150-mL flask containing 50 mL of a liquid MEB medium for five days. Then, the mycelium was harvested by filtration and washed successively with sterile Milli-Q water. The genomic DNA was extracted from the fungal cells using a Dneasy Plant Mini Kit (QIAGEN, Hilden, Germany). The purity and quantity of the DNA samples were estimated using the optical density ratio of A260/A280. The molecular identification was carried out with the protocol suggested by Daâssi et al. [27]. The primers used for the amplification were ITS1 (5-TCCGTAGGTGAACCTGCGG-3) and (3-TCCTCCGCTTATTGATATGC-5). BlastN analysis was used for the resulting sequences (www.ncbi.nlm.nih.gov/BlastN) (accessed on 11 March 2021). The organisms were identified based on the subjected sequences in the databases showing the highest identity. Multiple sequence alignment was achieved using Clustalmega between the selected subjected sequences and the query ITS sequences of the isolated strains [28]. A phylogenetic tree was inferred using the neighbour-joining method (NJ) [29] in the MEGA11 program, with bootstrap values based on 1000 replicates [30]. The sequences were deposited in GenBank.

### 2.7. Soil Batch Microcosm Reactors Assays

#### 2.7.1. Soil Preparation

Soil was prepared according to Tchobanoglous and Vigil [31]’s method. Soil moisture was adjusted to 70% of its field capacity (185 mL H_2_O/kg soil). Mineral solution MM (10.63 g (NH_4_)_2_SO_4_/kg soil and 0.70 g K_2_HPO_4_/kg soil) was used to adjust the C:N:P ratio to 100:15:1.

#### 2.7.2. Fungal Inoculum Preparation

The mycelial suspensions from the selected hydrocarbon-degrading exogenous ligninolytic (LB3) and (KB4) and indigenous (BKR1) fungi were prepared as described by Potin et al. [32]. Seven-day-old MEA-plate fungal cultures of the three selected isolates were washed with 15 mL of sterile physiological water to obtain the fungal suspension, then further filtrated through sterile glass wool to separate the mycelia from the conidia. The harvested conidia concentrations were estimated with a Thoma cell counting chamber. The conidia were added to the medium in calculated volumes to provide a final total spore concentration of 106 spores g soil^−1^.

#### 2.7.3. Soil Microcosm Treatments

All soil microcosms (SMs) with 100 g of contaminated soil were amended with nutrients at a C:N:P ratio of 100:15:1 and conducted at room temperature (25.5 ± 3.3 °C) for 60 days in triplicate.

Control soil microcosms (zero-day/native/not-inoculated) and test soil microcosms (zero-day/sterile/inoculated) for each fungal culture (mono- or mixed-culture) were set up for 60 days of treatment to evaluate the natural-attenuation-biostimulation (SMT1) versus biostimulation-bioaugmentation (SMT2) degradation treatments of the PHC-contaminated soil. A brief description of the SM’s experimental design is presented in Table 1.

The soil samples from each microcosm were collected manually with clean and sterilised (ethanol 70%) stainless steel spatulas on days 15, 30 and 60 for the analytic assessment of hydrocarbon degradation in the soil microcosms.

#### 2.7.4. Analysis of Soil Batch Microcosm Parameters

The residual TPH in contaminated soil [TPH]R was estimated using the EPA 3540C and EPA 821-B-94-004 methods [33,34].

Mechanical shaking, as described by Siddique et al. [35], with some modifications, was carried out for the extraction of the residual petroleum hydrocarbons from the contaminated soils. For each culture incubation period (15, 30 and 60 days), 10 g of soil samples was taken and the remaining [TPH]R were extracted from each SMs (SMT1/SMT2) using dichloromethane (DCM). The percentages of TPH were determined gravimetrically, and the results were expressed as percentages of the respective controls [25].

Biodegradation efficiency (%) was calculated using the formula given by Bishnoi et al. [36].

Biodegradation efficiency rate (%) R = [(TPH_0_ − TPHt))/TPH_0_] × 100 (1)

where TPH_0_ is the initial content of TPH (mg kg^−1^ soil) and TPHt is the TPH content at time x of (mg kg^−1^ soil).

The treated/extracted petroleum hydrocarbons were analysed using gravimetric analysis and gas chromatography with Agilent GC-MSD (6890N-5973) with the pole temperature kept at 80 °C for 4 min, then increased at a rate of 5 °C min^−1^ to 250 °C and maintained at 250 °C for 20 min.

The Biodegradation Rate Constant and Half-Life Time were calculated using Equation (2). The first-order biodegradation rate constant (k) was determined by evaluating the slope of the best-fit line on a plot of concentration vs. time using Equation (2).

C = C_0_ e^−kt^ (2)

where C_0_ is the initial concentration of hydrocarbons (mg TPH/kg soil) and C is the concentration of hydrocarbons at time (t) [37]. On the other hand, the half-life time (t1/2) was estimated from the derivative of Equation (3) [38].

T_1/2_ = ln2/k (3)

#### 2.7.5. Statistical Analysis

The obtained results were interpreted with one-way analysis of variance (ANOVA) to validate the statistical significance differences between groups using Tukey HSD (honest significant difference) with Statistica 11 software. The experimental design was a single factor with three levels and two replicates, having as a response variable the change in TPH concentration at a significance level (α) value of 0.05.

## 3. Results and Discussion

### 3.1. PHA-Contaminated Soil Sampling

The petroleum-contaminated soil samples were collected from oil well 7, located in Dammam City, Saudi Arabia, and maintained in plastic containers. The soil samples were collected from the surface layer (5–10 cm) at different spots around the petroleum pipeline spillage. The soil samples were mixed to form a composite sample that was used to represent areas of contamination in this study. The composite sample was sieved with a 6 mm grid before the soil microcosm treatments and a 2 mm grid for soil characterisation.

The primary physical properties of the soil were a dark colour, pH of 6.8 and average moisture content of 1.6 ± 0.2%. (Table 1).

The characterisation of the used petroleum-contaminated soil showed that the initial TPH (C10–C40 fraction) content of the soil was approximately 23,500 mg kg^−1^ (mg TPH/kg soil). Hydrocarbons of the chain length C15–C28 prevailed at 57.02%, followed by the fractions C29–C36 (31.36%) and C37–C40 (11.57%). Such characteristics are typical of hydrocarbons of natural origin, such as oil spills (Almutairi, 2022). A similar high content of TPH was shown in the study of Torres et al. [39], who reported a range of 51,550 to 192,130 mg kg^−1^ of TPH in soil samples collected from the production and oil exploration zones of the Tabasco state in Mexico.

Such characteristics are typical of hydrocarbons of natural origin, such as oil spills [40].

The soil sample contained an initial total aerobic heterotrophic bacteria population of 2.5 × 10^5^ colony-forming units per gram of soil.

### 3.2. Isolation and Selection of Hydrocarbon-Degrading Fungal Isolates

The primary identification of the ligninolytic fungal isolates was based on plate morphology, while the purity of the isolates was proofed using microscopic and ITS parameters. From the decayed wood samples, 10 pure wood-decomposing fungal isolates were isolated. From the investigated soil, two native-fungal strains designated as KBR8 and KBR1 and presenting the highest abundance and growth ability in the soil samples were examined using the soil-plate and soil-suspension isolation methods [24].

To select hydrocarbon-degrading fungal isolates, primary screening was conducted on culture plates based on the agar well diffusion method. In all Petri dishes, the highest growth diameter was around the mycelia of KBR1 and KBR8 (native soil isolates) and KB4 and LB3 (ligninolytic isolates), indicating good abilities to degrade diesel hydrocarbons among the isolated strains. However, KBR1-1 and KB2 showed tight haloes during fungal growth. In addition, the mixed culture consisting of the fungal consortium (KBR1 + LB3 + KB4) showed the highest growth diameter. Later, for a confirmatory assay of hydrocarbon degradation, the potentials of the isolated fungi were determined in Erlenmeyer flasks containing the investigated soil. The gravimetric determination of the residual hydrocarbons after biodegradation was performed by weighing the quantity of the petroleum hydrocarbons against the control.

**Figure 1:**
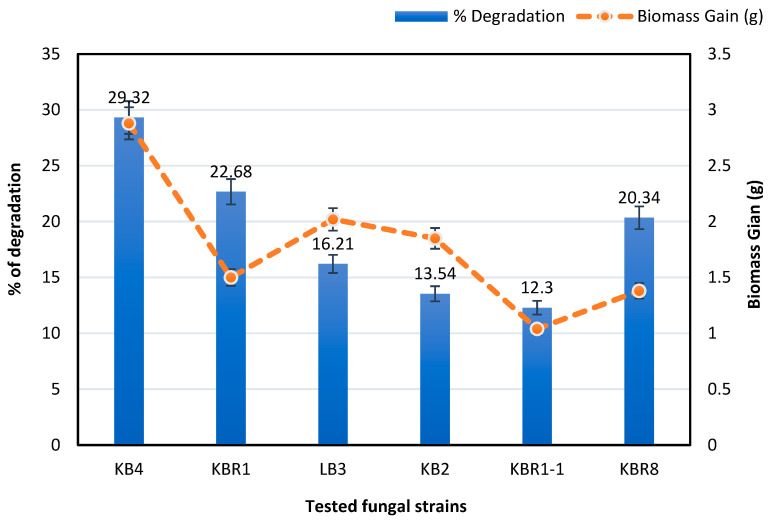
Percentage of petroleum degradation and biomass gain (g) of the tested isolated strains after 14 days on 5% crude oil in 100 mL MM. The data are the mean of three replicates, and the error bars represent the standard deviation.

Figure 1 shows an increase in the rates of fungal growth in the media containing petroleum-contaminated soil compared with the inoculated media (MM) with unpolluted soil. In culture flasks, the estimated crude oil degradation efficiency after 14 days demonstrated that the ligninolytic isolate KB4 showed the maximum ability to utilise crude oil, with the highest percentage of degradation at 29.32%, followed by the native isolates KBR1 and KBR8, which indicated 22.68% and 20.34% degradation, respectively.

Out of the twelve isolated strains investigated in the culture plate and in the flasks, six were recorded as petroleum hydrocarbon-degrading (KBR1, KBR8, KB4, LB3, KB2 and KBR1-1). Additionally, the mycelial growth and biomass gain profiles indicated that all the selected strains previously tested on the culture plates were able to grow and use petroleum hydrocarbons as a carbon source.

This result demonstrated the ability of the fungal strains to assimilate petroleum hydrocarbon molecules using diverse extracellular enzymes for their growth. In a related study on fungal strains isolated from hydrocarbon-polluted soil samples, Lotfinasabasl et al. [41] indicated that isolated fungi can be used in hydrocarbon bioremediation processes; however, their efficiency varies within the species and according to the fungi’s metabolic diversity. In this regard, Oboh et al. [42] reported the ability of *Aspergillus* sp., *Penicillium*, *Rhizopus* and *Rhodotorula* to grow on crude petroleum as the sole source of carbon and energy.

Among the 12 isolated fungi, the most interesting fungal strains in terms of crude oil degradation (LB3, KBR1, KBR8, KB2, KB4 and KBR1-1) were cultivated and run for molecular identification based on the analysis of the amplified nucleotide sequences of the nuclear ribosomal ITS1-5.8-ITS4 region.

A total of ten ligninolytic fungal isolates and two soil native strains were successfully isolated and then maintained as pure fungal cultures in MEA.

The purified isolates were tested for their ability to degrade petroleum hydrocarbons on culture plates and in flasks.

### 3.3. Molecular Identification and Phylogenetic Analysis of the Selected Petroleum Hydrocarbon-Degrading Fungi

The molecular identification of the strains was performed by the BLAST alignment tool of the National Center for Biotechnology Information (NCBI) database. Closely related sequences with similarities greater than 95% were obtained from the GenBank database. Based on the percentage of similarity (Table 2), all fungal isolates were attributed to the genus or even the species level according to Rosselló-Mora and Amman [43].

Based on the BLAST analysis, the isolated wood-decomposing fungi designated as KBR1-1 (strain 1), KB4 (strain 3), KB2 (strain 4) and LB3 (strain 6) were affiliated to the genera *Syncephalastrum* sp., *Paecilomyces formosus*, *Fusarium chlamydosporum* and *Coniochaeta* sp., respectively, while the indigenous petroleum-degrading fungi KBR1 (strain 2) and KBR8 (strain 5) belonged to *Aspergillus niger* and *tubingensis.*

**Figure 2:**
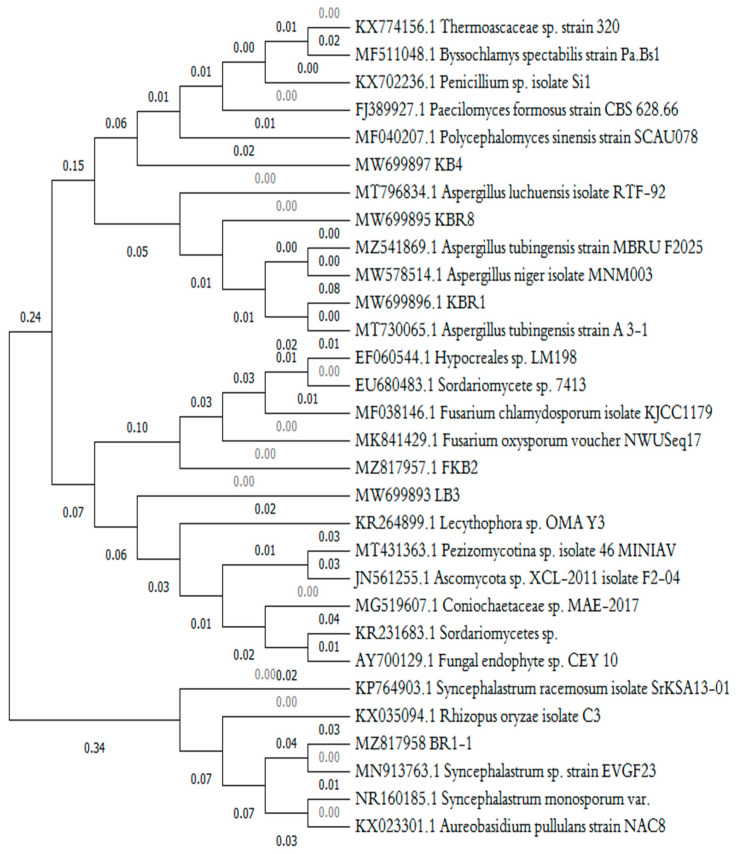
Phylogenetic tree for the hydrocarbon–degrading isolated fungi (exo- and indigenous isolates) and related sequences based on the BLAST alignment of ITS sequences. There were a total of 1484 positions in the final dataset. Evolutionary analyses were conducted in MEGA11 using the maximum composite likelihood method with bootstrap replicates.

Based on multiple alignments of the ITS sequences provided by the ClustalW program, phylogenic analysis was run to find the evolutionary relationships of the newly isolated fungi with previously characterised species (Figure 2).

The dendrogram obtained showed that the isolate LB3 (accession no. MW699893) clustered closely with *Coniochaetaceae* and *Sordariomycetes* sp. (99.68% identity) and with *Lecythophora* sp. (98.64% identity).

An analysis of the 18S rRNA genes of the genus Coniochaeta revealed that the taxon appeared as a monophyletic group related to *teleomorphs* of the genus *Lecythophora.*

According to Lopez et al. [44], *Lecythophora (Coniochaeta)* is a filamentous ascomycetous fungi that belongs to the Coniochaetaceae family and Sordariales order.

The strain KB4 (accession no. MW699897) showed 98.39% ITS identified with *Paecilomyces formosus*, *Thermoascaceae* sp. and *Penicillium* sp., and it was close to the genus Byssochlamys spectabilis (98.12%). The morphological traits of the fungus were determined to be affiliated with the isolate of the genus *Paecilomyces formosus* (yellow septate hyphae and unicellular conidia).

The genus Paecilomyces was first described by Bainier [45] as closely related to *Penicillium* and comprises only one species, P. variotii Bainier.

Accordingly, a previous study by Moreno-Gavíra et al. [46] reported that the genus *Paecilomyces* has yellowish septate hyphae with irregularly branched conidiophores and smooth walls. The unicellular conidia are in chains, and the youngest conidium is at the basal end.

The indigenous isolates (strain 2 and strain 5) were affiliated with the genus *Aspergillus* based on the BLAST analysis of the ITS sequences. In addition, phylogenetic analysis showed that the two indigenous isolates clustered in a clade comprised exclusively of *Aspergillus* species, with high bootstrap values for each branch. The ITS sequences of the fungal isolates were deposited to GenBank under the accession numbers MW699895 and MW699896 for KBR8 and KBR1, respectively.

The KBR1-1 isolate (strain 1) showed homology with close strains, including *Sordariomycetes* sp. (accession no. KR231683) (99.58% identity) and *Syncephalastrum racemosum* (accession no. KP764903.1) (98.87% identity).

It can be inferred from the phylogenetic tree that the strain closest to KBR1-1 was *Syncephalastrum racemosum.* The related sequence corresponding to the strain KBR1-1 was deposited under accession no. MZ817958.

The KB2 isolate (strain 4) (accession no. MZ817957) showed 98.84% ITS identified with *Fusarium chlamydosporum.*

Wood-decay decomposing fungi possess the metabolic capacity to degrade petroleum hydrocarbons. The isolated and identified fungal species agree with several researchers [47,48] that reported the involvement of the presented fungal strains in this work in the degradation of petroleum hydrocarbons. Fungi are able to grow into a wide spectrum of pollutants and produce extracellular enzymes that can modify or decompose petroleum hydrocarbon structures [49].

### 3.4. Microcosms for Petroleum-Contaminated Soil Remediation

To study the petroleum hydrocarbon removal ability of the fungal strains, the TPH in each SM system was recorded at different periods during the microcosm treatments assay. A brief description of the SM’s experimental design is presented in Table 3.

**Figure 3:**
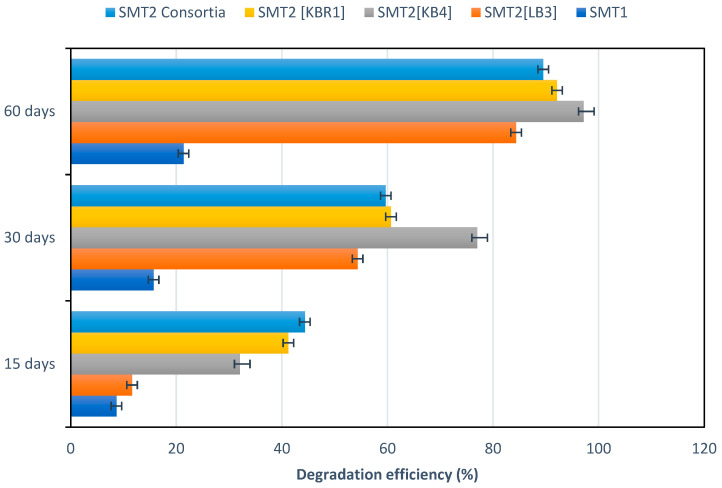
Degradation efficiency (%) in the soil microcosm for each treatment against the control microcosm.

According to Figure 3, TPH decreased by 41 ± 1.59% in SMT2 [KBR1], while a rapid decrease of 44 ± 1.8% was recorded in SMT2 [KBR1 + LB3 + KB4] after 15 days of incubation, in contrast to 11 ± 1.23% in SMT2 [LB3] and just 8 ± 0.15% in the control (SMT1). As depicted in Figure 3, an increase in TPH-biodegradation rates compared to SMT1 was observed in SMT2 microcosms, though biodegradation patterns were different depending on the added fungal species in single or mixed cultures. Additionally, the degradation rates for the fungal treatment process in SMT2 were higher than those for SMT1 (control: natural-attenuation biostimulation); values ranged from 1.43 to 5.12-fold those of the control during 15 days of incubation.

After 60 days of culture incubation, the highest rate of TPH degradation was recorded in SMT2 [KB4] at approximately 97 ± 2.54%, followed by SMT2 [KBR1] and then SMT2 [KBR1 + LB3 + KB4] with 92 ± 1.83% and 84 ± 2.21%, respectively. TPH biodegradation rates were close for all fungal treatments, but significantly improved in the function of the incubation period.

Statistical analysis demonstrated that there were significant differences (*p* < 0.05) in all soil microcosms, as follows: SMT2 [KBR1] (*p* = 0.0283), SMT2 [KB4] (*p* = 0.0442), SMT2 [KBR1 + LB3 + KB4] (*p* = 0.0188) and SMT2 [LB3] (*p* = 0.00254).

In the same context, Al-Hawash et al. [47] indicated that the hydrocarbon degradation rate could be enhanced with a longer incubation period during treatment.

As shown in Figure 3, the degradation efficiency in the soil microcosms is dependent on the treatment time, the used fungal species and the individual or mixed cultures. The presented results agreed with those of Hernández-Adame et al. [50] and Medaura et al. [20], who reported the improvement of the THP removal rate in the soil microcosm by fungal bioaugmentation.

Additionally, the differences between SMT1 and SMT2 indicate that bioaugmenting soil with wood-decay fungal species can critically influence the TPH-degradation rates compared to the conventional biostimulation strategy.

The soil microcosm augmented with the indigenous strain KBR1 (*Aspergillus niger*) showed a good degradation rate (approximately 92 ± 1.83%). This result was consistent with that of AI-Hawash et al. [47], who revealed the ability of the indigenous isolated *Aspergillus* sp. *RFC-1* to biodegrade several petroleum hydrocarbons such naphthalene, phenanthrene and pyrene at 51.8%, 84.6% and 50.3%, respectively.

The kinetics of TPH during the soil microcosm treatments demonstrated a rapid decrease of hydrocarbons in SMT2 [KBR1 + LB3 + KB4] compared with other SMs inoculated by a single strain. This result highlighted the essential role of the co-occurrence of different microbial species to enhance biodegradation yields. In this regard, Feng et al. [51] suggested that a combination of species with diversified metabolic activities is recommended to achieve the complete degradation of TPHs. In the co-culture, such species utilise intermediates of degradation produced by another strain, leading to the complete degradation of the oil [52].

Additionally, previous studies associated the importance of the incubation time with PAHs bioavailability. Thus, hydrocarbon compounds may have been adsorbed on soil particulars that made them difficult to remove. Daâssi and Almaghrabi [5] reported that TPHs-degrading microorganisms could mineralise or degrade petroleum hydrocarbon compounds depending on the rate of their accessibility and transfer to the fungal cell and their uptake and metabolism by microorganisms. Table 4 illustrated the results for all soil-microcosm processes conducted, and degradation rates are presented from highest to lowest.

The SM amended by the strain *Paecilomyces formosus (MW699897*) [KB4] showed the best degradation constant rate (0.059 days^−1^) and significantly lower half-life times (11.65 days), followed by *Aspergillus niger (MW699896)* [KBR1], the fungal mixed culture and *Coniochaeta* sp. *(MW699893)* [LB3]. The process of the natural attenuation that occurred in SMT1 yielded the lowest removal constant rate (4.01 × 10^−3^/day) and the longest half-life time (173.0779 days). These findings demonstrated that single fungal culture is more effective than the mixed culture. In line with our study, Obire et al. [53] claimed that a single fungal culture was better than mixed cultures to overcome the problem of competitiveness between the microbial communities. Conversely, some studies reported that the co-occurrence of microbial consortia had advantages over single species in degrading hydrocarbon mixtures [54].

It is generally established that the degradation efficiency average is influenced by various mechanisms during fungal treatment, including strain species, bioavailability, metabolic activities and the absorption of PHs into the hyphae surface. Literature studies have reported that the major factors affecting PHs biodegradation are subdivided into biotic and abiotic factors [5]. Additionally, the bio-remediate potential of microbes against petroleum contaminants relies on their specific metabolic activities [49].

### 3.5. GC-MS Analysis for Soil Microcosms

**Figure 4:**
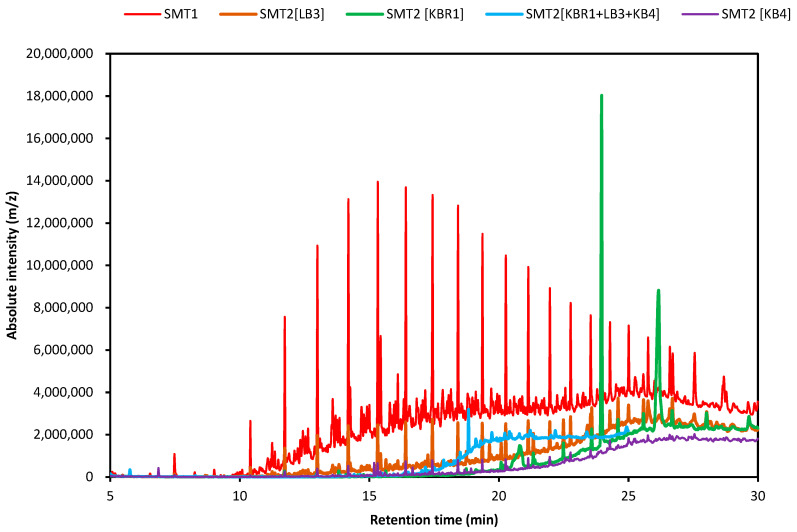
GC-MS superposed chromatograms of the residual hydrocarbons extracted from the soil microcosm treatment systems: control SMT1 (red), SMT2 [LB3] (brown), SMT2 [KB4] (violet), SMT2 [KBR1] (green) and SMT2 [KBR1 + LB3 + KB4] (blue).

The remaining PHs compounds were extracted and characterised by GC-MS for each SM treatment system after 60 days of culture incubation. Figure 4 illustrates the superposed profiles of PHs in the degrading soil microcosms using GC-MS analysis.

PHs remained in the soil microcosms but showed a decrease in the areas of major peaks compared with the control SMU, which suggested the degradation of the main compounds. Meanwhile, the appearance of new peaks in these samples indicated the breakdown of products or presumed metabolites. The control system (SMU-unsterile soil) represented the biotic effect of the native microbial communities in the contaminated soil and was incubated in the same experimental conditions as the treated SMs. The best degradation efficiency was obtained in SMT2 [KB4], followed by SMT2 [KBR1], then SMT2 [KBR1 + LB3+ KB4] and SMT2 [LB3].

**Figure 5:**
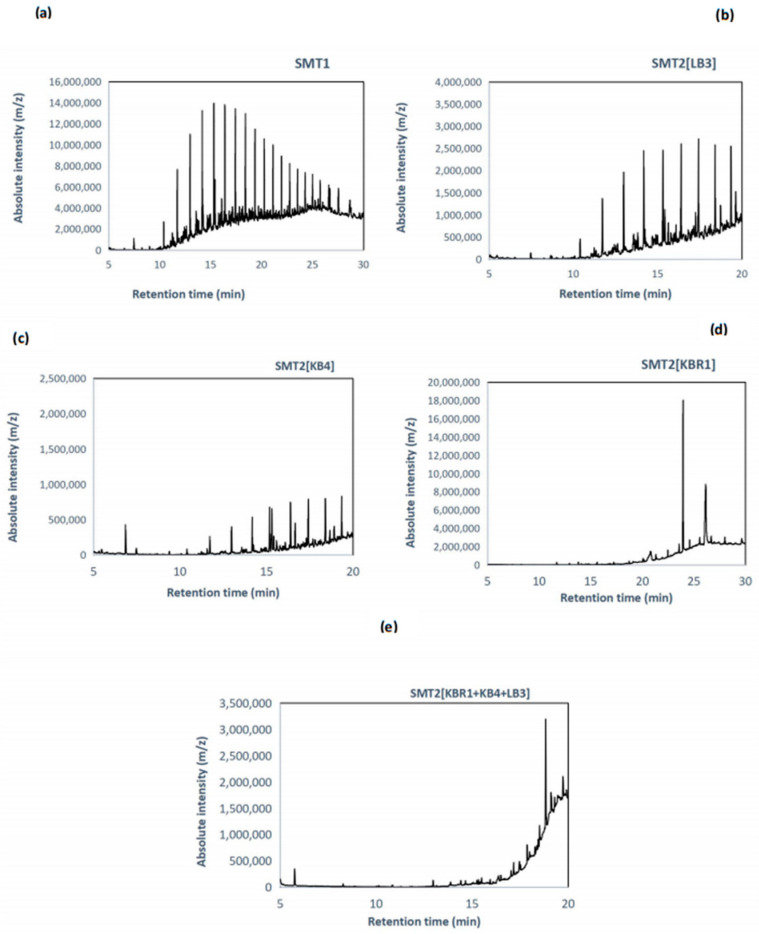
GC-MS chromatograms of the residual hydrocarbons extracted from the soil microcosm treatment systems: (**a**) SMT1, (**b**) SMT2 [LB3], (**c**) SMT2 [KB4], (**d**) SMT2 [KBR1] and (**e**) SMT2 [KBR1 + LB3 + KB4].

As seen in Figure 5, chromatograms revealed a significant reduction in the intensity of PHs peaks after SM treatment with fungal bioaugmentation (Figure 5b–d) compared with the control (Figure 5a).

GC-MS analysis performed after biodegradation showed that the biodegradation patterns of the petroleum hydrocarbon fractions in SMs treated by a single strain and SMs treated by mixed species were markedly different throughout time compared with the control microcosm. The GC-MS profiles demonstrated the efficiency of the newly isolated fungal strains in remediating petroleum-contaminated soil in the microcosm systems.

GC-MS was performed for the confirmation of PHCs biodegrading and identification of the volatile degradation products. The presented GC-MS profiles are similar to those reported by Al-Hawash et al. [47], using indigenous oil-degrading fungi affiliated to the genus *Penicillium.* However, the degrading compounds are mostly related to the microbial metabolic pathway.

Further quantitative and qualitative identifications of the main compounds in the extracted PHCs were conducted for different SM treatment systems (Table 5).

The data presented in Table 5 show that the abundant hydrocarbon fractions identified in the extracted PHCs from the control system (SMU) were C15–C28, followed by C29–C36 and C37–C40 hydrocarbon chain lengths.

Comparing the control system SMU with all the SM treatment systems, it is clearly showed that the fractions C29–C36 and C37–C40 were completely removed in the treated system SMT2 [KBR1 + KB4 + LB3], which constituted mixed culture *Coniochaeta* sp. (LB3), *Paecilomyces formosus* (KB4) and the native strain *Aspergillus niger*, whereas, despite its decreasing, those fractions were still persistent in the other SMT systems augmented by the monoculture of the same strains.

In this regard, many scientists have reported that mixed populations with overall broad enzymatic capacities are required to degrade complex structures of hydrocarbons. El-Aziz Arma et al. [55], in a related study on single and mixed fungal cultures, have reported similar findings.

Additionally, the system SMT2 [KBR1], employing the indigenous strain (*Aspergillus* sp.), was able to fully degrade the fraction C37–C40 and remove C29–C36 effectively (few amounts were recorded) after 60 days of culture incubation.

Furthermore, a varying number of biodegraded compounds of PHs was obtained due to the action of various microbial groups. Medaura et al. [20] reported that fungal bioaugmentation resulted in a higher biodegradation of total petroleum hydrocarbons (TPH) and of high molecular weight PHs than with biostimulation. TPH (C14–C35) decreased by 39 ± 1.99% in bioaugmented microcosms vs. 24 ± 1.31% in biostimulated microcosms.

Based on our results and on the scientific literature, biodegradation in petroleum-contaminated soil may be affected by biotic factors including the strains’ genera and their metabolic diversity, and further by abiotic factors such as environmental factors and hydrocarbons’ bioavailability for biological actions. In previous studies, the degradation ability of different genera from different habitats was shown to make their catabolic potential even more versatile to transform persistent organic compounds into inert and non-toxic molecules [49].

Overall, these results highlight the potential of ligninolytic fungi as well as soil indigenous fungi in the biodegradation or removal of petroleum hydrocarbons.

The GC-MS results provided further evidence of PHs’ degradation by ligninolytic fungal isolates. Our results seem to pave the way for further investigation to decipher the exact mechanism by which the fungal strain degrades PHs in the contaminated soil.

## 4. Conclusions

The present study revealed that exogenous fungal ligninolytic isolates and indigenous fungal flora collected from petroleum-contaminated soil samples hold promise for effective PHs bioremediation, notably, *Aspergillus niger*, *Aspergillus tubingensis*, *Syncephalastrum* sp., *Paecilomyces formosus*, *Fusarium chlamydosporum* and *Coniochaeta* sp. Further optimised research studies, scale-up and phytotoxicity assays are required to achieve an efficient degradation mechanism and to return treated soil to productive agroecosystems.

## Figures and Tables

**Table 1:** Physicochemical analysis of the petroleum-contaminated soil.

Property	Value
Physical Parameters	
Moisture Content (%)	1.6
pH	6.8
Total Petroleum Hydrocarbon (TPH)	
C6–C9 Fraction (mg kg^−1^)	<10
C10–C14 Fraction (mg kg^−1^)	<50
C15–C28 Fraction (mg kg^−1^)	13,400
C29–C36 Fraction (mg kg^−1^)	7370
C37–C40 Fraction (mg kg^−1^)	2720
C10–C40 Fraction (sum) (mg kg^−1^)	23,500
Volatile Organic Compounds-BTEX	
Benzene (mg kg^−1^)	0.2
Ethylbenzene (mg kg^−1^)	0.5
Meta- and Para-Xylene (mg kg^−1^)	0.5
Ortho-Xylene (mg kg^−1^)	0.5
Volatile Organic Compounds—Surrogates	
1.2-Dichloroethane-D4 (%)	100
Toluene-D8 (%)	99.3
4-Bromofluorobenzene (%)	95.9

**Table 2:** Molecular identification of petroleum hydrocarbon-degrading fungal isolates.

Number of Isolates	Code of Isolate	Origin of Isolate	Max Identity (%)	Strains of Closed Match (Accession Number)	Identification	Accession Number	Phylum
Strain 1	KBR1-1	Wood	99.58%	*Syncephalastrum* sp. *EVGF23* (MN913763.1)	*Syncephalastrum* sp.	MZ817958	Zygomycota
Strain 2	KBR1	PHs-Contaminated Soil	95.54%	*Aspergillus niger MNM003* (MW578514.1)	*Aspergillus niger.*	MW699896	Ascomycota
Strain 3	KB4	Wood	98.39%	*Paecilomyces formosus CBS 628.66* (FJ389927.1)	*Paecilomyces formosus*	MW699897	Ascomycota
Strain 4	KB2	Wood	98.84%	*Fusarium chlamydosporum isolate KJCC1179* (MF038146.1)	*Fusarium chlamydosporum*	MZ817957	Ascomycota
Strain 5	KBR8	PHs-Contaminated Soil	98.88%	*Aspergillus niger* (MW142509.1)	*Aspergillus tubingensis*	MW699895	Ascomycota
Strain 6	LB3	Wood	99.68%	*Coniochaetaceae* sp. *MAE-2017* (MG519607.1)	*Coniochaetaceae* sp.	MW699893	Ascomycota

**Table 3:** Soil microcosms treatments: experimental design.

Inputs	Soil Microcosms Treatments	
	SMT1	SMT2
Native soil (g)	+	−
Sterile Soil (g)	−	+
Nutrients C:N:P ratio of 100:15:1	+	+
Water	+	+
Fungal strains		
*Aspergillus niger* [MW699896] KBR1	−	+
*Coniochaeta* sp. [MW699893] LB3	−	+
*Paecilomyces formosus* [MW699897] KB4	−	+
*Fungal consortium culture:* KBR1 + LB3 + KB4	−	+

SMT1—unsterile: Microcosms formed by untreated, unsterile soil to assess biotic degradation (natural-attenuation biostimulation). SMT2—sterile: control microcosms made by air-drying untreated contaminated soil (sterile soil) to assess the abiotic losses of hydrocarbons. SMT2 [KBR1]: Biostimulation-bioaugmentation: soil amended and inoculated with selected native fungus (KBR1). SMT2 [LB3]: Biostimulation-bioaugmentation: soil inoculated with selected ligninolytic hydrocarbon-degrading fungus, LB3. SMT2 [KB4]: Biostimulation-bioaugmentation: soil inoculated with selected ligninolytic hydrocarbon-degrading fungus, KB4. SMT2 [KBR1 + LB3 + KB4]: biostimulation-bioaugmentation: soil inoculated with the co-culture (KBR1 + LB3 + KB4).

**Table 4:** Removal rate of TPH degradation, constant of rate and the half-life time (t_1/2_) for all soil-microcosm treatments after 60 days of incubation.

Removal Rate of TPH (%)	TPH Removal Rate (%)	k (1/Day)	t_1/2_ (Day)
SMT2 [KB4]	97.17933 ± 1.15	5.95 × 10^−2^ ± 1.71 × 10^−6^	11.65542
SMT2 [KBR1]	95.12733 ± 1.23	5.04 × 10^−2^ ± 2.1 × 10^−7^	13.76417
SMT2 [KBR1 + LB3 + KB4]	89.487 ± 2.04	3.75 × 10^−2^ ± 0.7 × 10^−6^	18.46294
SMT2 [LB3]	84.361 ± 1.65	3.09 × 10^−2^ ± 4.3 × 10^−7^	22.41499
SMT1	21.36 ± 2.35	4.01 × 10^−2^ ± 1.3 × 10^−7^	173.0779

[KB4] = *Paecilomyces formosus* (MW699897); [KBR1] = *Aspergillus niger* (MW699896); [LB3] = *Coniochaeta* sp. (MW699893).

**Table 5:** Main compounds identified in the dichloromethane-extracted PHs in the different soil microcosm treatment systems.

SMT1					
RT	Area%	Height%	Compounds	Molecular Formula	Fractions
11.735	5.34	1.81	Heptadecane	C_17_H_36_	C15–C28
12.995	7.64	1.98	Heptadecane	C_17_H_36_	
14.191	8.89	1.93	Heptadecane	C_17_H_36_	
15.326	9.2	1.82	Eicosane	C_20_H_42_	
15.436	3.43	2.34	Eicosane	C_20_H_42_	
16.407	8.75	1.81	Eicosane	C_20_H_42_	
17.15	1.15	5.02	Heptadecane, 2-methyl	C_18_H_38_	
17.439	8.5	2.07	Eicosane	C_20_H_42_	
18.142	0.83	5.11	2-methylhexacosane	C_27_H_56_	
18.423	7.84	1.88	Eicosane	C_20_H_42_	
19.366	6.7	1.87	Eicosane	C_20_H_42_	
20.268	5.93	1.89	Eicosane	C_20_H_42_	
21.136	5.46	1.9	Tetracosane	C_24_H_50_	
21.969	4.44	1.83	Hexatriacontane	C_36_H_74_	C29–C36
22.771	3.88	2.01	Hexatriacontane	C_36_H_74_	
23.544	3.16	1.82	Hexatriacontane	C_36_H_74_	
24.289	2.68	1.89	Hexatriacontane	C_36_H_74_	
25.011	2.4	1.96	Hexatriacontane	C_36_H_74_	
25.76	2.08	2.18	Tetrapentacontane	C_54_H_110_	C37–C40
27.558	1.7	3.27	2-methylhexacosane	C_27_H_56_	C15–C28
SMT2 [LB3]					
11.733	4.12	1.71	Heptadecane	C_17_H_36_	C15–C28
12.991	6.07	1.85	Heptadecane	C_17_H_36_	
14.184	7.16	1.76	Heptadecane	C_17_H_36_	
15.32	7.13	1.81	Heneicosane	C_21_H_44_	
15.432	2.54	2.3	Eicosane	C_20_H_42_	
16.401	7.28	1.78	Heneicosane	C_21_H_44_	
17.432	7.2	1.8	Eicosane	C_20_H_42_	
18.417	6.71	1.77	Eicosane	C_20_H_42_	
19.359	6.18	1.79	Eicosane	C_20_H_42_	
20.263	5.69	1.75	Eicosane	C_20_H_42_	
21.131	5.3	1.89	Eicosane	C_20_H_42_	
21.329	3.07	1.94	Cyclononasiloxane, octadecamethyl-	C_18_H_54_O_9_Si_9_	
21.964	4.31	1.86	Eicosane	C_20_H_42_	
22.492	4.03	1.94	Cyclononasiloxane, octadecamethyl-	C_18_H_54_O_9_Si_9_	
22.766	4.4	1.8	Hexatriacontane	C_36_H_74_	C29–C36
23.539	3.37	1.89	Tetracontane	C_40_H_82_	C37–C40
23.584	4.65	1.9	Cyclononasiloxane, octadecamethyl-	C_18_H_54_O_9_Si_9_	C15–C28
24.6	4.17	1.97	Cyclononasiloxane, octadecamethyl-	C_18_H_54_O_9_Si_9_	
25.582	3.19	2.25	Cyclodecasiloxane, eicosamethyl-	C_20_H_60_O_10_Si_10_	
26.696	3.43	2.5	Cyclononasiloxane, octadecamethyl-	C_18_H_54_O_9_Si_9_	
SMT2 [KB4]					
6.858	4.67	2.02	Cyclopentasiloxane, decamethyl-	C_10_H_30_O_5_Si_5_	C10–C14
12.99	4.12	1.73	Hexadecane	C_16_H_34_	C15–C28
14.184	5.61	1.78	Heptadecane	C_17_H_36_	
15.184	7.13	1.74	Methyl 13-methyltetradecanoate	C_16_H_32_O_2_	
15.318	6.85	1.83	Heneicosane	C_21_H_44_	
16.4	7.51	1.73	Heneicosane	C_21_H_44_	
16.669	4.14	1.86	Hexadecanoic acid, methyl ester	C_17_H_34_O_2_	
17.431	7.44	1.88	Eicosane	C_20_H_42_	
18.417	6.84	1.89	Eicosane	C_20_H_42_	
18.667	1.69	2.89	Methyl stearate	C_19_H_38_O_2_	
19.359	6.9	1.74	Eicosane	C_20_H_42_	
20.262	6.15	1.89	Eicosane	C_20_H_42_	
21.13	5.67	1.93	Eicosane	C_20_H_42_	
21.962	4.66	1.95	Eicosane	C_20_H_42_	
22.558	2.31	5.05	Bumetrizole	C_17_H_18_ClN_3_O	
22.765	4.8	1.84	Eicosane	C_20_H_42_	
23.539	3.56	1.71	Tetrapentacontane	C_54_H_110_	C37–C40
24.284	4.34	1.6	1-Eicosanol, 2-hexadecyl	C_36_H_74_O	C29–36
25.005	2.79	2.02	Hexatriacontane	C_36_H_74_	
25.753	2.82	1.96	Hexatriacontane	C_36_H_74_	
SMT2 [KBR1 + KB4 + LB3]					
5.758	6.34	2.1	Cyclotetrasiloxane, octamethyl-	C_8_H_24_O_4_Si_4_	C6–C9
17.869	6.65	1.51	Cyclononasiloxane, octadecamethyl	C_18_H_54_O_9_Si_9_	C15–C28
18.826	42.13	1.77	1-benzylindole	C_15_H_13_N	
19.108	7.45	2.56	Cyclodecasiloxane, eicosamethyl	C_20_H_60_O_10_Si_10_	
19.727	8.31	2.36	Cyclononasiloxane, octadecamethyl	C_18_H_54_O_9_Si_9_	
20.23	5.38	2.17	13.17.21-Trimethylheptatriacontane	C_7_H_14_	C6-C9
20.408	6.45	1.94	Cyclodecasiloxane, eicosamethyl	C_20_H_60_O_10_Si_10_	C15–C28
21.209	6.1	2.8	Tetracosamethyl-cyclododecasiloxane	C_24_H_72_O_12_Si	
22.178	6.44	2.94	Tetracosamethyl-cyclododecasiloxane	C_24_H_72_O_12_Si	
23.394	4.75	3.62	Tetracosamethyl-cyclododecasiloxane	C_24_H_72_O_12_Si	
SMT2 [KBR1]					
20.8	1.39	5.44	Silane, diethylheptyloxyoctadecyloxy	C_29_H_62_O_2_Si	C29–C36
22.489	2.59	2	Cyclodecasiloxane, eicosamethyl-	C_20_H_60_O_10_Si_10_	C15–C28
23.581	3.21	2.08	Tetracosamethyl-cyclododecasiloxane	C_24_H_72_O_12_Si	
23.966	58.04	2.78	1.3-Benzenedicarboxylic acid, bis(2-ethylhexyl) ester	C_24_H_38_O ·	
24.598	3.15	2.04	Tetracosamethyl-cyclododecasiloxane	C_24_H_72_O_12_Si	
25.581	2.58	2.18	Tetracosamethyl-cyclododecasiloxane	C_24_H_72_O_12_Si	
26.163	22.36	7.93	Propanoic acid, 3.3’-thiobis-, didodecyl ester	C_30_H_58_O_4_S	
26.688	2.56	2.88	Tetracosamethyl-cyclododecasiloxane	C_24_H_72_O_12_Si	
28.015	2.51	3.53	Tetracosamethyl-cyclododecasiloxane	C_24_H_72_O_12_Si	
29.654	1.61	4.06	Tetracosamethyl-cyclododecasiloxane	C_24_H_72_O_12_Si	

## Data Availability

An earlier version of our manuscript was presented as “pre-print” under the ref.no [10869/v1.] in “research square” according to https://doi.org/10.21203/rs.3.rs-1086969/v1 [56].

## Supplementary Materials

The following supporting information can be downloaded at: https://www.mdpi.com/article/10.3390/microorganisms11051351/s1, Table S1: A table of the physicochemical analysis of the petroleum-contaminated soil with general comments and the used methods (DOCX).
