# Supplementary material for: Petroleum-Degrading Fungal Isolates for the Treatment of Soil Microcosms
Source: Microorganisms. 2023 May 22;11(5):1351. doi: 10.3390/microorganisms11051351 (PMC10224032; doi:10.3390/microorganisms11051351)
Supplement: Supplementary file 1 [file microorganisms-11-01351-s001.zip › microorganisms-2390288-supplementary.pdf]

Table S1. Physicochemical Analysis of the PAHs-contaminated soil with details

ANALYTICAL RESULTS					
PHYSICAL PARAMETERS					
Compound	Cas#	Technique/ Method Reference	[a]LOR	Unit	Value
^ Moisture Content (dried @ 103°C)	-----	-	1.0	%	1.6
pH	-----	-			6.8
TOTAL PETROLEUM HYDROCARBON (TPH)					
C6 - C9 Fraction	----	USEPA 5030/8260	10	mg kg^{-1}	<10
C10 - C14 Fraction	----	USEPA 3510/8015 GC/FID	50	mg kg^{-1}	<50
C15 - C28 Fraction	----		100	mg kg^{-1}	13400
C29 - C36 Fraction	----	-	100	mg kg^{-1}	7370
C37 - C40 Fraction	----	-	50	mg kg^{-1}	2720
^ C10 - C40 Fraction (sum)		-	100	mg kg^{-1}	23500
VOLATILE ORGANIC COMPOUNDS - BTEX					
Benzene	71-43-2	USEPA 5030/8260	0.2	mg kg^{-1}	0.2
Ethylbenzene	100-41-4		0.5	mg kg^{-1}	0.5
meta- & para-Xylene	108-38-3 106-42-3		0.5	mg kg^{-1}	0.5
ortho-Xylene	95-47-6		0.5	mg kg^{-1}	0.5
Toluene	108-88-3		0.5	mg kg^{-1}	0.5
VOLATILE ORGANIC COMPOUNDS - SURROGATES					
1.2-Dichloroethane-D4	17060-07-0	USEPA 5030/8260	0.2	%	100
Toluene-D8	2037-26-5		0.2	%	99.3
4-Bromofluorobenzene	460-00-4		0.2	%	95.9

[a] LOR= Limit of reporting

Surrogate Control Limits		Recovery Limits (%)	
Compound	Cas#	Low	High
VOLATILE ORGANIC COMPOUNDS - SURROGATES			
C COMPOUNDS - SURROGATES 1.2-Dichloroethane-D4	17060-07-0	53	134
4-Bromofluorobenzene	460-00-4	59	127
Toluene-D8	2037-26-5	60	131

General comments

The analytical procedures used by the Life Sciences Division have been developed from established internationally recognized procedures such as those published by the USEPA. APHA. AS. NEPM. FDA/BAM. AOAC. ISO etx. In house developed procedures are employed in the absence of documented standards or by client request.

Brief Method Summaries

The analytical procedures used by the Life Sciences Division have been developed from established internationally recognized procedures such as those published by the USEPA. APHA AS. NEPM. FDA/BAM. AOAC. ISO etc. In house
developed procedures are employed in the absence of documented standards or by client request .

Analytical Methods	Method	Matrix	Method Descriptions
Moisture Content / Dry matter	EA055	SOIL	In-houes; A gravimetric procedure based on weight loss over a 12 hour drying period at 103-105 degrees C. This method is compliant with NEPM (2013) Schedule B(3) (Method 102)
Total Petroleum Hydrocarbon (Semivolatile)	EP071	SOIL	US EPA 8015B (M) GC/FID; Sample extracts are analysed by Capillary GC/FID and quantified against alkane standards over the range C10 - C40
TPH Volatiles / BTEX US	EP080	SOIL	EPA 8260 P&T/GC/MS; Extracts are analysed by Purge and Trap. Capillary GC/MS. Quantification is by comparison against an established 5 point calibration curve.
Miscellaneous Tests *	*S-MISC	SOIL	Miscellaneous Tests

Preparation Methods	Method	Matrix	Method Descriptions
Methanol Extraction of Soils for Purge and Trap	ORG16	SOIL	(USEPA SW 846 - 5030A) 5g of solid is shaken with surrogate and 10mL methanol prior to analysis by Purge and Trap - GC/MS
Tumbler Extraction of Solids (Option B - Non-concentrating)	ORG17B	SOIL	In-house. Mechanical agitation (tumbler). 10g of sample. Na2SO4 and surrogate are extracted with 20mL 1:1 DCM/Acetone by end over end tumble. The solvent is transferred directly to a GC vial for analysis

`*` symbol preceding any method indicates laboratory or subcontractor non-accredited test. In the case when a procedure belonging to an accredited method was used for non-accredited matrix. would apply that the reported results are non-accredited.
